# Pretreatment Low Serum Sodium as a Prognostic Factor for Patients with Esophageal Cancer Treated with Radiotherapy or Chemoradiotherapy

**DOI:** 10.1155/2022/4586729

**Published:** 2022-03-21

**Authors:** Jianchao Lu, Yi Wang, Mei Lan, Jiahua Lv, Tao Li, Lei Wu, Qifeng Wang, Jinyi Lang

**Affiliations:** Department of Radiation Oncology, Sichuan Cancer Hospital and Institution, Sichuan Cancer Center, School of Medicine, University of Electronic Science and Technology of China, Radiation Oncology Key Laboratory of Sichuan Province, Chengdu, China

## Abstract

Low serum sodium levels have been associated with poor prognoses for several cancers. However, the prognostic value of low serum sodium levels in esophageal carcinoma (EC) has not been well elucidated. We examined the prognostic value of low baseline serum sodium levels before radiotherapy or chemoradiotherapy for EC patients. A retrospective analysis of data from EC patients who received radiotherapy or chemoradiotherapy at a single cancer center was performed. Patients were divided into low serum sodium level (≤140.0 mmol/L) or high serum sodium level (>140.0 mmol/L) groups according to the median pretreatment serum sodium level. The Kaplan–Meier model and Cox proportional hazards model were used for survival analyses. The 5-year progression-free survival (PFS) and overall survival (OS) rates in the whole group were 16.9% and 21.8%, respectively. The PFS and OS rates of patients in the low serum sodium levels group were significantly lower than those in the high serum sodium levels group (*p* < 0.001). A similar association between PFS/OS and sodium levels was observed in the treatment subgroups. The univariate analysis showed that low serum sodium levels, Karnofsky performance status (KPS), clinical N stage, tumor site, clinical stage, and treatment mode were the influencing factors of OS. Multivariate analyses indicated that low baseline serum sodium levels were an independent prognostic marker of poor PFS (HR, 1.744; 95% CI, 1.248-2.437; *p* = 0.001) and OS (hazard ratio (HR), 2.125; 95% confidence interval (CI), 1.555-2.904; *p* < 0.001). Pretreatment levels of low serum sodium could be a new and helpful serum biomarker of the prognosis of EC patients receiving radiotherapy or chemoradiotherapy.

## 1. Introduction

Esophageal carcinoma (EC) is a common malignancy, ranking seventh in terms of prevalence and sixth in terms of mortality worldwide. It is well established that the incidence of EC and the mortality of EC patients vary among geographic areas in China, with some areas reporting an incidence rate up to 116.87 per 100,000 and a mortality rate of 95.76 per 100,000 [1]. The 5-year overall survival (OS) rate ranges from 15% to 25% worldwide [2]. Squamous cell carcinoma is the main pathological type and is one of the most prevalent and lethal types, with a mortality rate of almost 90% in China [2]. Although there have been significant developments in the pathology of EC and comprehensive treatments are available, patient outcomes need to be improved. Little attention has been focused on biomarkers that can predict the prognosis. Therefore, it is necessary to explore biomarkers as new prognostic indicators that could potentially guide clinical practice.

Hyponatremia is a common serum disorder of electrolytes that frequently occurs in patients with solid tumors [3–5]. Numerous studies have revealed that hyponatremia is related to poor prognoses for several solid tumors, including tumors of the bladder, lung, breast, liver, colon, and rectum, as well as head and neck [3, 6–9]. Serum sodium is widely recognized as a time-saving, economical, repeatable, and routine prognostic biomarker that can predict patient prognosis. Previous studies have shown that hyponatremia is an adverse event that emerges in 16% to 59% of EC patients who undergo chemotherapy or chemoradiotherapy [10–12].

To our knowledge, few studies have investigated the relationship between baseline serum sodium levels and survival of EC patients. The incidence and prognostic value of serum sodium levels before any treatment for EC patients have been underestimated. Therefore, we retrospectively reviewed 271 EC patients to address this issue. Pretreatment serum sodium concentrations were assessed in association with OS and progression-free survival (PFS). Additionally, the association of pretreatment serum sodium levels with OS and PFS after different types of procedures was analyzed.

## 2. Materials and Methods

Our study received approval from the Ethics Committee of Sichuan Cancer Hospital, and informed consent was exempted by the ethics committee. Patients with biopsy-proven EC who had undergone radical radiotherapy or chemoradiotherapy were enrolled at the Sichuan Cancer Hospital between March 2006 and October 2016.

The inclusion criteria were as follows: Karnofsky score ≥ 70 points, pathologically confirmed EC, underwent radical radiotherapy or chemoradiotherapy, no history of malignant disease, and underwent a routine blood test and biochemical examination within 1 week before any treatment. In contrast, the exclusion criteria were as follows: severe medical disorders, underwent treatment at other institutions, had not received radiation doses of above 50.4 Gy, and insufficient information or incomplete laboratory or clinicopathological parameters.

Radical radiotherapy was administered for more than five weeks with a cumulative dose of 50.4 to 60.0 Gy. Some of these patients underwent radiation therapy concomitant with platinum-based chemotherapy. The patients were stratified by treatment modalities and divided into low and high serum sodium groups according to the median value.

### 2.1. Data Collection and Definition

The pretreatment serum sodium concentration was acquired from venous blood within one week before radiotherapy or chemoradiotherapy and was conventionally available from the hospital's laboratory at Sichuan Cancer Hospital. The baseline serum sodium concentration was recorded as the median value. A low serum sodium level was defined as ≤140.0 mmol/L, and a high serum sodium level was defined as >140 mmol/L. Clinical factors and demographic data were retrospectively collected manually from the medical records. Clinicopathological data included the date of diagnosis, age, sex, Karnofsky score, tumor histology, tumor site, staging, serum sodium level, and follow-up information. All patients were pathologically confirmed to have EC. The pathological stage was reassessed based on the TNM classification system, as defined by the American Joint Committee on Cancer (8th edition). All cases were examined and followed up at least every three months during the first two years, every six months for the following one to three years, and every 12 months after five years. The information collected during the follow-up period included the results of physical examinations, endoscopic examinations, imaging, and laboratory tests. Prognostic information included PFS and OS. PFS and OS were defined as the length of time between the initiation of treatment and the date of either death or disease progression and the length of time between the initiation of treatment and the date of all-cause death, respectively.

### 2.2. Statistical Analysis

Sample characteristics were compiled using descriptive statistics. Pearson's chi-square (*χ*^2^) test and Student's *t*-test (the Mann–Whitney *U* test was performed if the data were not normally distributed) were used to assess the relationship between the patient's tumor characteristics and serum sodium levels. The Kaplan–Meier model and Cox proportional hazards model were used for survival analyses. Univariate and multivariable Cox proportional hazards models were employed to assess factors associated with esophageal cancer prognosis. After assessing the *p* value (*p*) from the univariate model, variables with *p* < 0.1 were introduced into multivariable Cox proportional hazards models. Statistical analyses were conducted using SPSS software version 24.0 (SPSS Inc., Chicago, IL, USA).

## 3. Results

### 3.1. Patient Characteristics

In total, 271 patients of biopsy-proven EC stage I-IVB were enrolled at Sichuan Cancer Hospital from March 1, 2006, to October 31, 2016. However, fifteen patients with the following criteria were excluded from the analysis: recurrent EC that had been previously treated (*n* = 4); patients with metastases to distant organs (*n* = 5); patients who stopped any of the treatments (*n* = 1); noncompletion of the treatment (*n* = 2); and incomplete available information, such as follow-up data and clinicopathological or laboratory parameters (*n* = 3). Therefore, only 256 patients were eligible for further analysis, including 92 patients who underwent radiotherapy alone and 164 patients who underwent chemoradiotherapy (Table 1). The average age of all patients was 64 years (range, 35-92 years). There were 196 men and 60 women with a Karnofsky score ≥ 70 points. According to the reference range of serum sodium levels (135-145 mmol/L), the rate of hypernatremia (>145 mmol/L) and the rate of hyponatremia (<135 mmol/L) for the enrolled patients were 1.6% and 6.3%, respectively.

**Figure 1:**
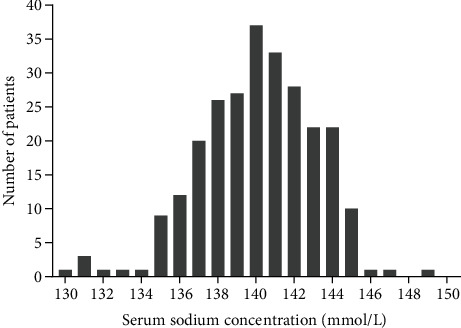
Graph showing distribution of serum sodium concentration in 256 patients.

The last follow-up was completed in February 2018, and the median follow-up time was 35 months (range, 12.4-81.2 months). The median baseline serum sodium level was 140.0 mmol/L (range, 130.0-149.0 mmol/L) (Figure 1). Of the 256 patients, 123 fell into the high serum sodium group and 133 fell into the low serum sodium group. Of all the patients, 76.6% were male, 47.3% were older than 65 years, and 9.4% had a Karnofsky performance score of 70 points. In addition, 99.6% of the patients had squamous cell carcinoma, and 43.8% of tumors were localized in the middle of the esophagus. None of the clinicopathological characteristics (age, sex, Karnofsky score, tumor site, histopathology, T and clinical N stage TNM classification, and type of treatment regimen) was statistically associated with serum sodium concentrations assessed before treatment (Table 1).

### 3.2. Survival Outcomes

**Figure 2:**
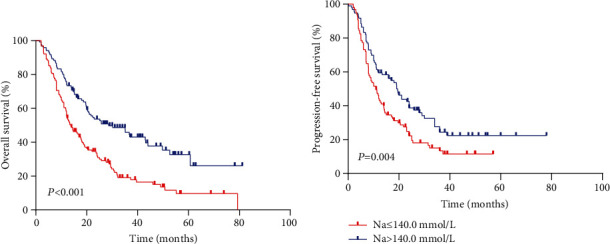
(a) Kaplan–Meier survival curves of the serum sodium concentration at the median cutoff value (140.0 mmol/L) are shown (*p* < 0.001). (b) Kaplan–Meier survival curves of the serum sodium concentration at the median cutoff value (140.0 mmol/L) are shown (*p* = 0.004).

The median PFS and OS for all evaluable patients were 12.6 months and 16.9 months, respectively. The overall 5-year PFS and OS rates were 16.9% and 21.8%, respectively. Analysis results indicated that outcomes in the high serum sodium group were better than those in the low serum sodium group (Figures 2(a) and 2(b)) (OS *p* < 0.001, PFS *p* = 0.004). The 5-year PFS rates were 22.4% and 11.5%, and the 5-year OS rates were 32.7% and 11.7% in the high and low serum sodium groups, respectively.

**Figure 3:**
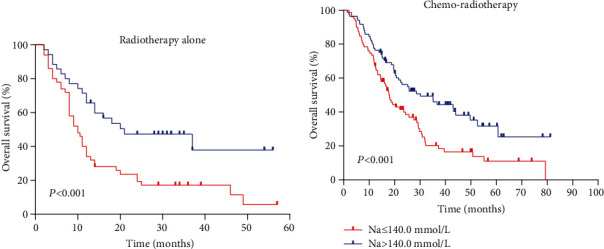
(a) Kaplan–Meier survival curves for OS according to the serum sodium concentration in the radiotherapy alone subgroup. (b) Kaplan–Meier survival curves for OS according to the serum sodium concentration in the chemoradiotherapy subgroup.

Next, we performed a subgroup analysis according to treatment modes. An analysis of OS with radiotherapy alone and chemoradiotherapy (Figures 3(a) and 3(b)) showed that the high serum sodium group also had improved outcomes compared to the low serum sodium group (both *p* < 0.001).

### 3.3. Association of Serum Sodium Concentration with Inflammatory Response

**Figure 4:**
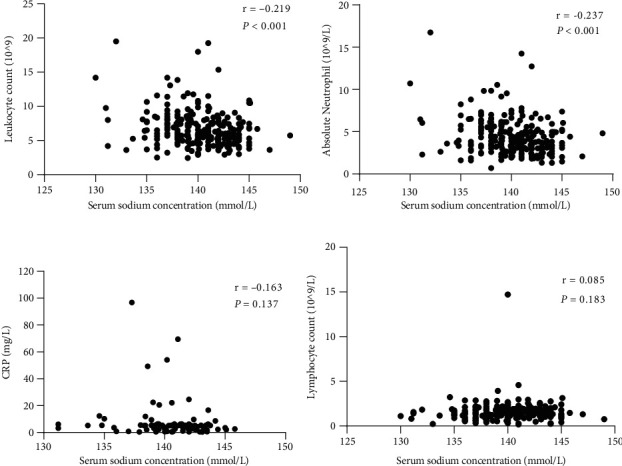
The association of serum sodium concentration with blood indicators. (a) Scatterplot for relationship between sodium and leukocyte count. (b) Scatterplot for relationship between sodium and neutrophil. (c) Scatterplot for relationship between sodium and CRP. (d) Scatterplot for relationship between sodium and lymphocyte.

Furthermore, we observed a significant negative correlation between low pretreatment concentration of serum sodium and neutrophil and leukocyte levels (Figures 4(a) and 4(b)).

The relationships among clinicopathological factors and serum sodium concentration are shown in Table 2. These data clearly show that the neutrophil count was negatively associated with the baseline serum sodium level (*p* < 0.001), and a significant negative correlation was observed between the serum sodium level and the neutrophil-to-lymphocyte ratio (NLR) (*p* < 0.001). The trend of the negative association between C-reactive protein (CRP) levels and serum sodium concentrations was not statistically significant (*p* = 0.137) (Figure 4(c)). In addition, there was no correlation between serum sodium levels and lymphocyte counts (*p* = 0.183) (Figure 4(d)). Alternatively, there was no association between the neutrophil count and CRP level (*p* = 0.325).

### 3.4. Univariate and Multivariate Analyses

The univariate analysis showed that low serum sodium levels (≤140.0 mmol/L), Karnofsky performance status (KPS), clinical N stage, tumor site, clinical stage, and treatment mode were the influencing factors of OS (*p* < 0.05) (Table 3). Similarly, the prognostic factors that significantly and independently affected PFS were the clinical stage of the carcinoma (*p* = 0.002), Karnofsky performance score (*p* = 0.022), and serum sodium level (*p* = 0.005) (Table 4).

In the multivariate Cox proportional hazards regression models, the serum sodium levels (*p* < 0.001), Karnofsky score (*p* < 0.001), treatment type (*p* < 0.001), clinical stage (*p* = 0.017), and clinical N stage (*p* < 0.001) were identified as significant and independent unfavorable outcome prognostic factors (Table 3).

According to the results of stratified and multivariate Cox proportional hazards model analysis for PFS and OS, the low serum sodium group's PFS (*p* = 0.001; HR [95% CI], 1.744 [1.248-2.437]) and OS (*p* < 0.001; HR [95% CI], 2.125 [1.555-2.904]) hazards ratios were higher than those of the high serum sodium group. In other words, patients with baseline serum sodium concentrations ≤ 140.0 mmol/L had a lower survival rate than those with serum sodium levels > 140.0 mmol/L (Figures 2(a) and 2(b), Tables 3 and 4). This suggests that a low baseline serum sodium level is an independent and significant prognostic factor for poor survival.

## 4. Discussion

In the present study, we assessed the prognostic value of baseline factors and pretreatment serum sodium levels by performing a retrospective analysis of 256 EC patients. The incidences of hypernatremia and hyponatremia (routinely classified in the laboratory) for untreated patients were 1.6% and 6.25%, respectively. This observation is in accordance with the previously reported incidence of hyponatremia (range, 4–47%) [5, 13].

We confirmed that EC patients with baseline serum sodium levels ≤ 140.0 mmol/L had significantly shorter survival than those with high serum sodium levels (*p* < 0.001). Similar results were obtained by performing a subset analysis of the different treatment groups (*p* < 0.001 for both comparisons). In addition, Cox proportional hazards model analysis showed that the risk of mortality in the low serum sodium level group was 2.15 times that of the high serum sodium level group, and the risk of disease progression was 1.744 times.

Our study demonstrated that the decrease in serum sodium concentrations before initial treatment was inversely associated with the outcomes of EC patients. A previous study indicated that the reduction in serum sodium levels (per 3 mmol/L decrease) was significantly related to a 19% increased risk of death [9]. However, the mechanisms underlying the development of low serum sodium levels remain unclear, particularly for EC patients. Recently, an increasing number of biological markers have been identified that may have an essential role in cancer patients with serum sodium level abnormalities. Poor appetite, weight loss, and cachexia are commonly found in malignant diseases and particularly in EC patients who may develop malnutrition. Decreased oral intake, in particular, leads to decreased serum sodium levels.

Moreover, high tumor burden, gastrointestinal fluid loss, and renal fluid loss may disturb the serum sodium balance, which induces vomiting and diarrhea, resulting in hypovolemic hyponatremia [14]. Abnormal secretion of antidiuretic hormone in tumor patients may increase the reabsorption of water from the distal renal tubule and decrease plasma osmolality [15, 16]. A systemic inflammatory response is likely an additional factor that alters the serum sodium levels. Furthermore, a vicious cycle of the exacerbation of the production of inappropriate antidiuretic hormones might exist between inflammatory states and serum sodium levels [17].

We evaluated the association between low serum sodium levels and inflammation. Concomitantly, we found that the pretreatment serum sodium concentration was inversely associated with CRP, leukocyte count, neutrophil count, and NLR; however, it was positively associated with lymphocyte count in EC patients. Notably, no statistical differences were observed in the association between pretreatment serum sodium and CRP levels or lymphocyte counts. Similary, no significant correlation was found between the neutrophil count and CRP levels. This may be attributed to variations in detection levels over time and incomplete CRP data. A previous study consistently showed that white blood cell count was the risk factor in EC patients [11]. It has also been reported that CRP, IL-6, IL-1*β*, and neutrophil counts are associated with hyponatremia. Furthermore, this observation has been reported for nonmalignant diseases [18]. Secretion of antidiuretic hormones from neurons is promoted by the proinflammatory cytokines IL-6 and IL-1*β* in the internal milieu [19, 20], and the inflammatory response is further promoted through inflammasome activation in macrophages, which might be induced by cell swelling-stimulated osmolality [21]. Increasing evidence has suggested that ion channels and pumps not only have a major role in maintaining intracellular and extracellular pH and regulating membrane potential stability but also have critical roles in the regulation of cell migration [22]. These findings also suggest that decreased serum sodium levels could be a prognostic marker, although the underlying molecular mechanism remains unclear.

In the present study, the 5-year OS for patients treated with radiotherapy was 17.5%, which was lower than the 5-year OS of 34% reported by Lin et al. [23]. This could be partially due to the fact that 36.2% of the patients recruited for their study had stage I or II tumors, whereas only 3.5% of the patients in our study had stage I or II. In the present review, the 5-year OS rate for chemotherapy and radiotherapy (23.6%) was significantly higher than that for radiotherapy alone (17.5%) (*p* = 0.01), which is similar to other findings [24, 25] and in accordance with the Radiation Therapy Oncology Group 8501 data [26]. At 5 years of follow-up, the OS for combined modality therapy was 26% compared with 0% after radiation therapy. Based on previous clinical data, chemoradiation is a standard strategy for EC patients.

The prognostic value of the tumor site in EC patients has been previously reported. We found that cervical EC had a better prognosis than carcinoma at other sites. However, further multifactor analysis did not show statistical significance. This may have been caused by confounding factors that could have influenced our results, such as patient selection.

It is commonly recognized that the N stage has emerged as a prognostic marker of outcomes of EC patients. Concomitantly, performance status has been determined to be a predictor of outcomes. Previous studies revealed that better performance status is associated with better tolerance of chemoradiation in EC patients [27]. Our study found that patients with a Karnofsky performance status of 70 had a worse prognosis than those with a Karnofsky performance status of 80 to 90 (*p* < 0.001).

To the best of our knowledge, this study is the first to demonstrate the predictive and prognostic values of baseline serum sodium concentrations of EC patients treated with radiotherapy alone or chemoradiotherapy. Moreover, serum sodium is regularly, quickly, and economically obtained during routine blood tests. Nevertheless, the causal associations among low serum sodium levels, ion channels and pumps, and inflammation for EC patients remain unclear and require further study.

Our study had several limitations. First, it was a retrospective, single-center analysis that spanned almost 10 years. Additionally, the sample size was relatively small. Finally, kinematic data of serum sodium were not collected.

## 5. Conclusions

We confirmed that low pretreatment serum sodium levels are associated with poorer OS and PFS for patients treated with radiotherapy alone or chemoradiotherapy. Serum sodium concentrations have the potential to be a significant prognostic factor of EC patients. However, a prospective large-scale study of EC patients is needed to fully understand the prognostic role of low serum sodium levels.

## Figures and Tables

**Table 1:** Patient and tumor characteristics in relation to serum sodium levels.

Characteristic	Total *N* (%)	Sodium levels (mmol/L)		*p* value
		Low serum sodium group *N* (%)	High serum sodium group *N* (%)	
*N*	256	133 (52.0)	123 (48.0)	
Age (years)		64.38 ± 10.61	64.47 ± 10.25	0.942^∗^
Sex				0.127
Male	196 (76.6)	107 (80.5)	89 (72.4)	
Female	60 (23.4)	26 (19.5)	34 (27.6)	
KPS				0.184^†^
70	25 (9.8)	14 (10.5)	11 (8.9)	
80	134 (52.3)	74 (55.6)	60 (48.8)	
90	97 (37.9)	45 (33.8)	52 (42.3)	
Tumor sites				0.302^†^
Cervical	24 (9.4)	8 (6.0)	16 (13.0)	
Upper	76 (29.7)	42 (31.6)	34 (27.6)	
Middle	112 (43.8)	58 (43.6)	54 (43.9)	
Lower	44 (17.2)	25 (18.8)	19 (15.4)	
Histopathology				0.480‡
Squamous	255 (99.6)	133 (100)	122 (99.2)	
Nonsquamous	1 (0.4)	0 (0)	1 (0.8)	
T stage				0.123^†^
T2	30 (11.7)	18 (13.5)	12 (9.8)	
T3	138 (53.9)	75 (56.4)	63 (51.2)	
T4a	36 (14.1)	16 (12.0)	20 (16.3)	
T4b	52 (20.3)	24 (18.0)	28 (22.8)	
Clinical N stage				0.514^†^
N0	16 (6.3)	8 (6.0)	8 (6.4)	
N1	120 (46.9)	62 (46.6)	58 (47.2)	
N2	107 (41.8)	52 (39.1)	55 (44.7)	
N3	13 (5.1)	11 (8.3)	2 (1.6)	
M stage				0.624
M0	207 (80.9)	106 (79.7)	101 (82.1)	
M1	49 (19.1)	27 (20.3)	22 (17.9)	
Clinical stage				0.713^†^
IIB	9 (3.5)	3 (2.3)	6 (4.9)	
IIIA	15 (5.9)	7 (5.3)	8 (6.5)	
IIIB	127 (49.6)	69 (51.9)	58 (47.2)	
IVA	59 (23.0)	29 (22.8)	30 (24.4)	
IVB	46 (18.0)	25 (18.8)	21 (17.1)	
Treatment types				0.106
Radiotherapy	92 (35.9)	54 (40.6)	38 (30.9)	
Chemoradiation	164 (64.1)	79 (59.4)	85 (69.1)	

Abbreviation: KPS: Karnofsky performance status. ^∗^*t*-test. ^†^Mann–Whitney test; ^‡^Fisher's exact test. Other *p* values were tested by Pearson's chi-square (*χ*^2^) test. Serum sodium levels: low sodium: ≤140.0 mmol/L; high sodium: >140.0 mmol/L.

**Table 2:** Spearman's correlation analysis of interrelationships among hematological indicators.

Hematological Indices		r_s_	*p* value
Serum sodium	Leukocyte	-0.219	<0.001
	Neutrophil	-0.237	<0.001
	CRP	-0.163	0.137
	Lymphocyte	0.085	0.183
	NLR	-0.247	<0.001
Leukocyte	Neutrophil	0.910	<0.001
	CRP	0.186	0.088
	Lymphocyte	0.363	<0.001
	NLR	0.381	<0.001
Neutrophil	CRP	0.108	0.325
	Lymphocyte	0.088	0.167
	NLR	0.634	<0.001
CRP	Lymphocyte	0.068	0.533
	NLR	0.029	0.792

Abbreviation: NLR, neutrophil-to-lymphocyte ratio; CRP, C-reactive protein. r_s_: Spearman's correlation coefficient.

**Table 3:** Cox regression analysis of clinical characteristics of overall survival of EC patients who underwent radiotherapy alone or chemoradiotherapy.

	Univariate			Multivariate		
	HR	*p* value	95% CI	HR	*p* value	95% CI
KPS	2.207	<0.001^∗∗∗^	1.408-3.462	2.974	<0.001^∗∗∗^	1.862-4.751
70						
80-90						
Tumor sites						
Cervical	1 (reference)					
Upper	1.753	0.096	0.906-3.393			
Middle	1.979	0.034^∗^	1.052-3.723			
Lower	2.611	0.005	1.331-5.119			
Clinical N stage						
N0	1 (reference)			1 (reference)		
N1	1.964	0.112	0.855-4.512	3.617	0.003^∗∗^	1.529-8.558
N2-3	2.690	0.019^∗^	1.175-6.158	5.205	<0.001^∗∗∗^	2.175-12.455
Clinical stage	1.371	0.038^∗^	1.017-1.848	1.473	0.017^∗^	1.070-2.026
II-III						
IV						
Treatment models	0.665	0.011^∗^	0.487-0.910	0.475	<0.001^∗∗∗^	0.340-0.663
Radiotherapy						
Chemoradiotherapy						
Serum sodium	1.966	<0.001^∗∗∗^	1.448-2.669	2.125	<0.001^∗∗∗^	1.555-2.904
>140 mmol/L						
≤140 mmol/L						

Abbreviation: EC: esophageal carcinoma; HR: hazard ratio; CI: confidence interval; KPS: Karnofsky performance status. ^∗^*p* < 0.05. ^∗∗^*p* < 0.01. ^∗∗∗^*p* < 0.001.

**Table 4:** Cox regression analysis of clinical characteristics of progression-free survival of EC patients who underwent radiotherapy alone or chemoradiotherapy.

	Univariate			Multivariate		
	HR	*p* value	95% CI	HR	*p* value	95% CI
KPS	1.785	0.022^∗^	1.088-2.928	1.707	0.035^∗^	1.040-2.802
70						
80-90						
Tumor sites						
Cervical	1 (reference)					
Upper	1.444	0.225	0.798-2.615			
Middle	1.618	0.097	0.916-2.857			
Lower	2.11	0.018^∗^	1.136-3.922			
Clinical N stage						
N0	1 (reference)					
N1	1.623	0.298	0.652-4.038			
N2-3	2.113	0.105	0.855-5.220			
Clinical stage	1.689	0.002^∗∗^	1.220-2.338	1.846	<0.001^∗∗∗^	1.325-2.573
II-III						
IV						
Treatment models	0.892	0.617	0.571-1.395			
Radiotherapy						
Chemoradiotherapy						
Serum sodium	1.594	0.005^∗∗^	1.149-2.213	1.744	0.001^∗∗^	1.248-2.437
>140 mmol/L						
≤140 mmol/L						

Abbreviation: EC: esophageal carcinoma; HR: hazard ratio; CI: confidence interval; KPS: Karnofsky performance status. ^∗^*p* < 0.05. ^∗∗^*p* < 0.01. ^∗∗∗^*p* < 0.001.

## Data Availability

Data used and analyzed during the current study will be available from the corresponding authors upon reasonable request.
